# Supplementary material for: In Vitro-Based Production of Virus-Tested Babaco (Vasconcellea x heilbornii, syn. Carica pentagona) in Ecuador: An Integrated Approach to an Endangered Crop
Source: Plants (Basel). 2023 Jul 6;12(13):2560. doi: 10.3390/plants12132560 (PMC10346516; doi:10.3390/plants12132560)
Supplement: Supplementary file 1 [file plants-12-02560-s001.zip › plants-2392444-supplementary.pdf]

Protocol S1. Protocol for *in vitro* propagation of babaco (*Vasconcellea x heilbornii)*

1. Prior to *in vitro* introduction, make weekly applications with 500 ml of streptomycin solution (150 mg/L of distilled water) directly at the base of the stem (Figure 1A) and 5 ml of a contact fungicide based on Maneb (C4H6MnN2S4), Zineb ($C_4H_6N_2S_4Zn$), and cooper sulphate (CuSO4 · 5H2O) (2.5 gr/L of distilled water) on the foliage surface (Figure 1B). This step should be done for a minimum of 8 weeks.

2. After 8 weeks in the greenhouse, take the main branch and make a single cut to extract a ~ 5 cm long segment of the stem (explant) (Figure 1C). Remove the leaves and transfer the explant to the laboratory avoiding direct exposure to the environment.

3. Place the explants in a container and wash gently with a solution of water and liquid hand soap using a ratio of 1:3 (soap:water) for about 3 minutes (Figure 1D).

4. Place the explants in a glass jar and cover with a piece of gauze pad held with a rubber band and remove the soap from the explants by allowing tap water to run on top of the gauze pad for 20 minutes (Figure 1E). Carefully, dump the water from the jar and take it to the laminar flow chamber.

5. Immerse the explants, sequentially, in a 70% ethanol solution (1 min), 2% chlorine solution (10 minutes) and sterile distilled water (1 min). Repeat the water rinse 4 times (Figure 1F).

6. Immerse the explants in a solution of povidone iodine (2.5 % v / v in distilled water) for 5 minutes. Remove the explants from the solution draining carefully to remove the excess. Let the explants drain overnight in a sterile container inside the laminar flow chamber.

7. Cut small (~1.5 cm long) fragments of its basal end with a previously sterilized scalpel and place it with a tweezers into a container having a mixture of 30 ml of Murashige and Skoog basal medium, Gamborg Basal Salts Mixture M0404, 30 g / L of sucrose, 7 g / L of agar at a pH of 5.7 (Figure 2B).

8. Keep the explants in a sterile environment for 30 days under controlled conditions: 16 hours of light at 25°C. Constantly monitor the explants during this time to corroborate their proper development and remove contaminated samples.

9. *Subculture 1*: transfer the developed buds to a container with 25 ml of MS medium supplemented with Thidiazuron (TDZ) (1mg/L) and indoleacetic acid (IAA) (0.5 mg/L). Keep them for 30 days under the same conditions described above (Figure 2C).

10. *Subculture 2*: transfer the new developed shoots from each explant into a new container with 25 ml of MS medium supplemented with 1 mg / L of TDZ and 0.5 mg / L of IAA (Figure 2C).

11. *Subculture 3*: transfer the shoots into a new container with 25 ml of MS medium supplemented with 6-benzcylaminepurine (BAP) (1 mg/L). *Note: the number of subcultures will depend on the number of shoots desired. It is not recommended to perform more than 5 subcultures* (Figure 2D).

12. Perform a final subculture by placing the new plants in a container with MS medium supplemented with indole butyric acid (IBA) (1 mg/L). Keep them for 30 days under the same conditions described above (Figure 2E).

13. Extract rooted plants and wash the roots with distilled water to remove medium residuals.

14. Transplant the *in vitro* rooted plants to a plastic tray with lid containing sterile LM-18 substrate supplemented with liquid medium ½ MS salts and 1 ml of contact fungicide solution (2.5 mg/L distilled water). Place the tray in an area whit shade net to avoid direct sunlight with a photoperiod of 12 hours of daylight and at a temperature of 25 °C. Keep the plants under these conditions for 30 days. During this period, fertilize the plants every two weeks by direct irrigation of the soil with a solution of liquid medium ½ MS supplemented with 2 mg / L of (IBA) with 0.5 mg / L of naphthaleneacetic acid (ANA).

15. Transfer the plants to plastic cups containing sterile LM-18 substrate supplemented with liquid ½ MS salts. Keep the plants for 60 days in an area with shade net to avoid direct sunlight with a photoperiod of 12 hours of daylight and at a temperature of 25 °C.

16. Water the plants using distilled water as needed. Once the plant has grown and reached ~15 cm height, transplant them to one-gallon plastic bags (Figure 2F).

Supplementary material Table S1. Primer information for the detection of babaco viruses.

	Primer information (5' – 3')				
**Virus**	**Forward**	**Reverse**	**Annealing temperature °C**	**Amplification product**	**Reference**
Papaya ringspot virus	GAGARGTAYATGCCGCGGTATGG	CGCATACCCAGGAGAGAGTGC	55	263	Quito-Avila et al. 2015
Babaco mosaic virus	GGATGCACTCATTACATCCAAGC	CCACTCCAAGGCTTCCATGAGC	57	647	Alvarez-Quinto et al. 2017
Babaco cheravirus-1	GCTTGTCATTAGCACGGCTAAC	GCAGGAAAGAGCGTCTGATCA	55 - 57	447	Cornejo-Franco et al. 2020
Babaco nepovirus -1	GGTATGCTCGACAGAGCATTGT	CCCTTCTACATTCCACAACCAC	55 - 57	269	Cornejo-Franco et al. 2020
